# Eco-friendly spectrofluorimetric determination of mirabegron in human plasma and tablets: greenness and blueness assessment

**DOI:** 10.1186/s13065-025-01653-5

**Published:** 2025-10-29

**Authors:** Sayed M. Derayea, Ebtehal F. Anwer, Deena A. M. Nour El-Deen

**Affiliations:** Analytical Chemistry Department, Faculty of Pharmacy, Minia University, Minia, Egypt

**Keywords:** Mirabegron, Erythrosine B, Fluorescence quenching, Real human plasma, Tablets formulations, Greenness, Blueness

## Abstract

The current article describes facile, selective and highly sensitive spectrofluorimetric methodology for the determination of Mirabegron (MBG). The approach originated from the drug’s reaction with Erythrosine B in an acidic buffered media leading to the production of a binary complex. This interaction caused a significant quenching in the intensity of the fluorescence of Erythrosine B at an emission wavelength ( ʎ_em_ ) of 550 nm, following excitation ( ʎ_ex_) at 520 nm. Key parameters of the procedure were optimized before constructing the calibration curve. A direct proportionality was obtained between the fluorescence quenching and MBG concentrations ranging from 0.05 to 1.2 µg/mL. Both limits of detection (LOD) and quantitation (LOQ) have been calculated to be 0.01 and 0.04 µg/mL, respectively. Implementing the demands set by the ICH, the developed technique was assessed to evaluate the level of accuracy, precision, and robustness. The suggested approach was employeds to determine the drug being studied in pharmaceutical tablets. Moreover, the suggested spectrofluorimetric method exhibited an elevated level of sensitivity, making it suited for accurately determining the mentioned drug in real plasma samples with outstanding recovery and a low relative standard deviation. The environmental sustainability of the proposed method was systematically evaluated through AGREE (Analytical Greenness Metric) and BAGI (Blue Applicability Grade Index) assessments, demonstrating superior eco-friendliness compared to conventional HPLC and electroanalytical techniques (AGREE score: 0.72 vs. 0.55–0.62). In addition to confirmation of the environmentally safety of the proposed approach, its blueness has been assessed using Blue Applicability Grade Index.

## Introduction

 The overactive bladder syndrome (OAB) is known as urinary urgency which escorted by urinary frequency as well as nocturia [1]. Mirabegron is the first ß_3_-adrenoceptor agonist class of drugs that approved for the treatment of this syndrome either alone or in combination with antimuscarinic drugs. It was approved for clinical use as Betmiga in Europe (2013). MBG acts by increasing the concentration of cyclic adenosine monophosphate in the bladder tissue resulting in the relaxation of the bladder [2, 3].

Mirabegron has been analyzed in the last year by some analytical methods such as spectrophotometric methods [4–9], spectrofluorimetric methods [10–15], electroanalytical methods [16–18] as well as HPLC techniques [19–28]. Chromatography-based methods show remarkable selectivity and have therefore been used to detect degradation products and to study drug pharmacokinetics. However, these methods need a long analysis time and involve the use of complex and expensive instruments. In addition, HPLC methods consume huge amounts of high-purity organic solvents which are expensive and pose significant risks to the environment. Furthermore, some of these chromatographic methods use very expensive detectors [26–28].

On the other hand, spectrofluorimetric techniques are very simple analytical tools that have high sensitivity and simple instruments. It could be tailored to be safe to the environment through careful choosing the reagents and solvents [29]. However, the reported spectrofluorimetric methods suffer certain limitations including; the use of multiple components reagents with indirect procedure [10], the use of highly harmful reagent [11], and heating for long time [12–15]. In addition, these spectrofluorimetric methods were applied for the analysis of dosage forms [10–15] and real plasma [12] or spiked human plasma [15]. Neither of these spectrofluorimetric methods were applied to estimate MBG in real human plasma. Consequently, the focus in the current work was directed toward developing a spectrofluorimetric approach that could surpass the previous restrictions with high sensitivity.

The ultimate goal of this research is to set up a simple and swift approach that could be used for assessing MBG in pharmaceutical formulations as well as human plasma. This could be attained by utilizing the ability of MBG to give an ion pair associated with erythrosine B. The complex formation was employed for developing a novel spectrofluorimetric for the determination of MBG based on the quenching impact of the drug on the native fluorescence of the dye. The proposed method is thought to be the first effort to determine MBG in real human plasma using a fluorescence-based approach in comparison to the reported methods [10, 11]. The newly developed method utilizes cost-effective reagent and solvent.

In addition, these substances are readily available in quality control and environmentally safe and these approaches get rid of the necessity for laborious extraction steps and hazardous organic solvents in addition to elaborate apparatus.

## Experimental

### Apparatus

JASCO FP_6200 spectrofluorometer (Japan) supported with 150-Watt Xenon lamp have been used throughout the present work. The instrument features a wavelength accuracy of ± 1.5 nm, a photomultiplier voltage of 700 V, and a spectral bandwidth of 5 nm for both excitation and emission. All measurements were performed using 10 mm quartz cells at 25 °C controlled by a Peltier thermostatic cell holder. In addition, a Shimadzu spectrophotometer (Japan) linked to Lab-solutions UV–Vis’s software (version 1.10) was also used.

### Chemicals and reagents

MBG powder has been generously supplied by Amoun Company in Cairo, Egypt with a purity (99.8%). It was used without any prior treatment. Betmiga ^®^ tablets, produced by Multipharma, Cairo, Egypt, is label to contain 50 mg MBG in each tablet. The purchase of Erythrosine B was made from (Sigma Aldrich, UK). Erythrosine B was dissolved in an aqueous solution to give a concentration of 0.016% w/v. A solution of acetate buffer was produced by combining certain volumes of 0.2 M solutions of acetic acid and sodium acetate. Methanol ethanol, acetone, acetonitrile sodium acetate and acetic acid were acquired from El Nasr Company, that is located in Cairo, Egypt.

### Standard drug solutions

As a first step, 10 mg of MBG was specifically added to a 100 mL volumetric flask and dissolved in methanol. This was carried out to prepare a stock solution with a concentration of 100 µg/mL. Next, a series of dilutions were performed to get the working solutions of 0.5–12.0 µg/mL. Finally, 1.0 mL of each working solution was taken and added to separate 10 mL volumetric flasks.

### Method’s procedure

#### General assay procedure

First, adequate amounts of MBG standard solution (0.5–12.0 µg/mL) were transferred into a sequence of 10.0 mL calibrated flasks. Next, 1.0 mL of acetate buffer (pH 3.5) was transferred, then 1.0 mL of erythrosine B dye (0.016% w/v) was added. In the final step, the flasks were diluted with distilled water, and the fluorescence was monitored at 550 nm upon excitation at 520 nm. The obtained intensity of fluorescence was subtracted from the reading of the blank which was treated similarly without using the drug solution.

#### Preparation of the pharmaceutical tablet solutions

For the purpose of analyzing the commercial pharmaceutical tablets, Betmiga ^®^ Tablets, firstly, ten particular tablets were taken, weighted precisely, finely grinded and mixed. Secondly, a certain number of milled tablets containing 10 mg of MBG was accurately weighed and dissipated in methanol via sonication for approximately 5 min. Filtration of this solution was performed into a 100 ml volumetric flask, which it was completed to the mark with methanol. This solution was subjected to dilution and a portion from it was analyzed using the procedure of the general assay mentioned previously.

#### Procedure for stoichiometry of the reaction

To determine the reaction stoichiometry between MBG and erythrosine B, Job’s method was performed. Solution of MBG as well as erythrosine B having the same concentration (1.8 × 10^− 4^ M) were prepared. Next, several solutions containing MBG and the dye in a complementary manner were mixed and followed by adding 1.0 mL of acetate buffer (pH 3.5). Distilled water was utilized to dilute these solutions to 10 mL and the absorbance was measured at 558 nm. Finally, blank experiments containing only the dye were set up and their absorbance were measured. The absorbance values of the solutions containing different drug concentrations were corrected as per their blank readings. The corrected absorbance was plotted versus the molar fraction of MBG to set up the Job`s plot.

#### Procedure for spiked human plasma

Any procedure, including the use of human plasma, was approved and carried out in agreement with the recommendations and guidelines of the research ethical committee, Faculty of Pharmacy, Minia University. Plasma was obtained from the blood bank, Minia University Hospital (Minia, Egypt). Then 1.0 mL of the plasma was spiked with 1.0 mL of MBG standard solution (50–1200 ng/mL). After that, 2 mL of acetonitrile was added as a protein precipitating agent, and the mixture underwent centrifugation at 4000 rpm for about 20 min. The supernatant was collected carefully and then subjected to analysis using the general procedure [30]. A blank sample was analyzed in the same manner using plasma samples free from the studied drug.

#### Preparation of human plasma samples for calibration standard and quality control

The preparation of calibration standard (CS) and quality control (QC) in plasma was performed by spiking 1.0 mL of plasma sample with 1.0 mL of standard solutions having different concentrations of the drug. The final concentrations of MBG in (CS) ranged from 50 to 1200 ng/mL, while in QC samples, they were 100, 500 and 900 ng/mL as at low, medium and high concentration levels. Plasma proteins were precipitated by adding 2 mL of acetonitrile and removed by centrifugation at 4000 rpm for 20 min. A portion of the clear supernatant was analyzed according to the general analytical procedure.

#### Procedure of real human plasma

Healthy volunteers administered two Betmiga ^®^ 50 mg tablets with a total dose of 100 mg, for 7 successive days. After five hours of the last dose, 5.0 mL of blood was collected and transferred into a heparinized centrifugation tube [30]. Then the content was centrifuged at 4000 rpm for 10 min and the clear plasma solution was moved to another tube and stored at 20 °C till the time of analysis. The supernatant was collected carefully and then subjected to analysis using the general procedure.

All plasma samples (real, CS and Q samples) were stored at 20 °C, and thawed at room temperature just prior to analysis.

##### Ethics approval

This work was approved by the Committee of Research Ethics in the Faculty of Pharmacy, Minia University, Minia, Egypt with approval number (MPEC (250604)).

## Results and discussion

**Fig. 1:**
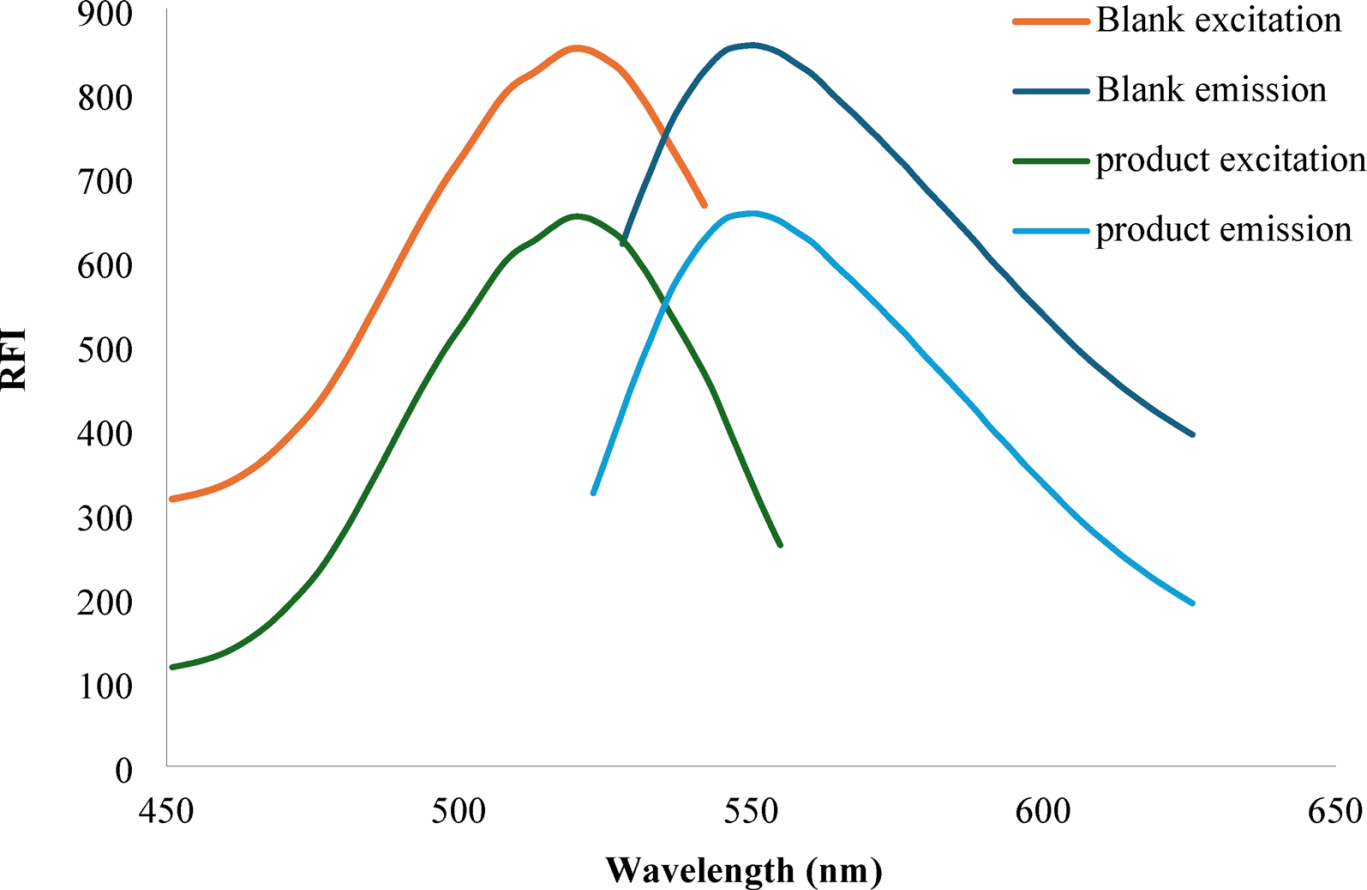
Excitation and emission spectra for erythrosine B alone (blank) and the reaction product between erythrosine B (0.016% w/v and mirabegron (0.5 µg/mL) in the presence of acetate buffer (pH 3.5)

Erythrosine B is one of the acidic xanthene dyes that exhibits strong fluorescence. In a mildly acidic environment, it can undergo an ion pair association process with amino group-containing compounds, resulting in formation of a stable complex. This reaction was used as a fluorometric approach for the determination of many pharmaceutical compounds [31]. The assay was based on quantifying the extent to which the drug under test could reduce erythrosine B’s native fluorescence. In the present study, fluorescence quenching can be quantified at an emission wavelength of 550 nm following excitation at 520 nm (see Fig. 1). The degree of fluorescence quenching was directly related to the concentration of MBG and provided a highly sensitive approach that could be applied for the analysis of MBG in real human samples.

### Optimization of the parameters of the reaction

To maximize the sensitivity of the method, the parameters of the reaction for the proposed procedure were carefully evaluated and optimized. This involved adjusting the pH, volume of acetate buffer, and volume of erythrosine B dye.

#### The effect of pH of the buffer

**Fig. 2:**
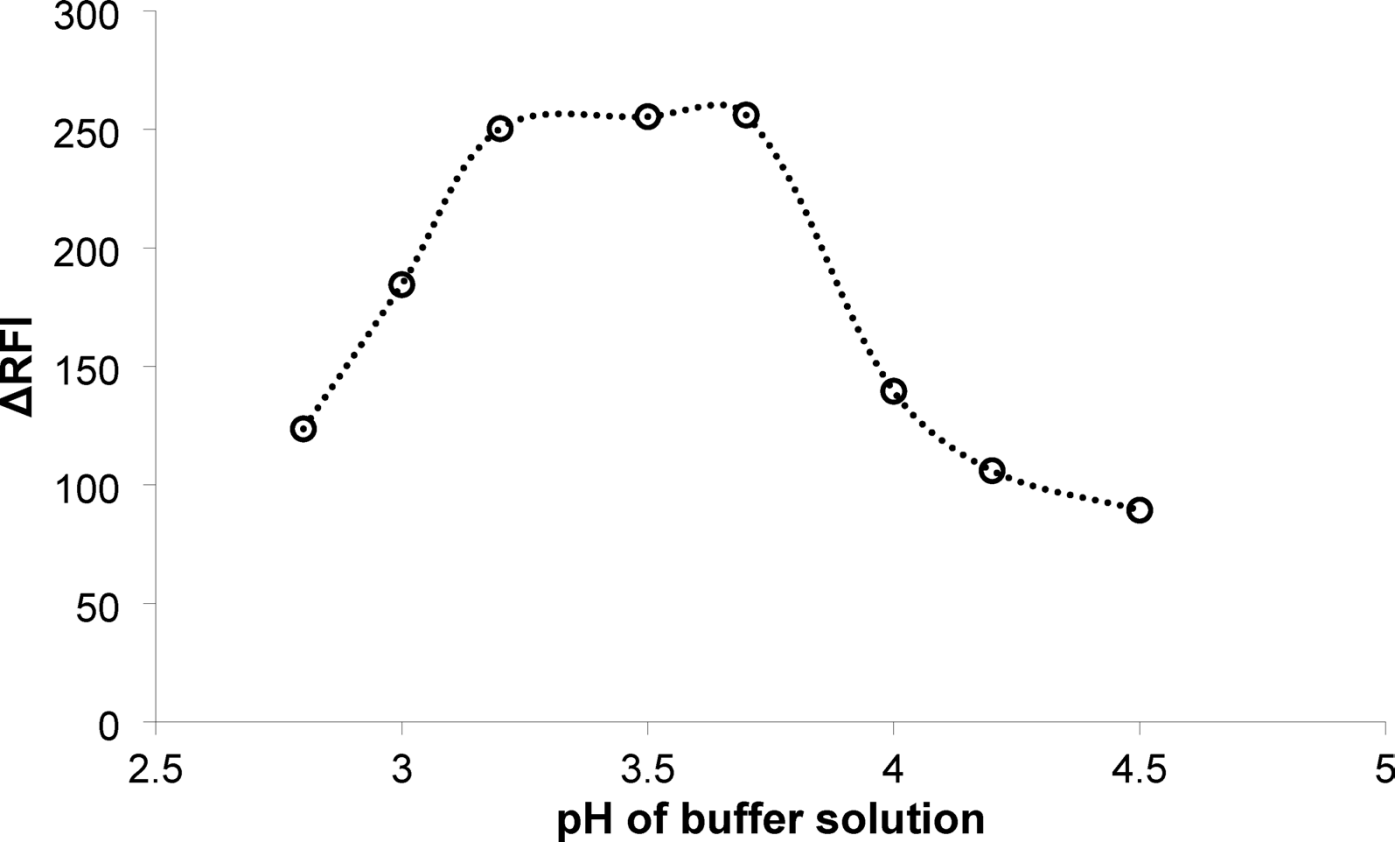
The effect of pH of acetate buffer on the difference in relative fluorescence intensity of the reaction product between mirabegron and erythrosine B

The ion pairing reaction of the present study has been shown to be strongly influenced by the pH of the reaction. Erythrosine B is an acid dye that only undergoes ion pairing in acidic conditions. In aqueous solution, the xanthine dye, erythrosine B, undergoes a two-step ionization reaction with pKa1 = 3.9 and pKa2 = 5.0 [32]. The ion pairing process is not suitable in a strongly acidic media with a pH lower than 2.9. Under these conditions, the hydroxyl moiety on the dye molecule would become protonated, resulting in a positive charge. The positive charges on both the drug and the dye cause repulsion between them, which prevents the creation of ion pair associate. Conversely, in a strongly alkaline medium, the secondary amine group of the medicine being investigated would not ionize effectively, resulting in the drug’s inability to produce a complex with the anionic dye. Thus, the highly basic environment was also unsuitable for the process of complexation. Consequently, both the highly acidic and highly alkaline environments were omitted from the current investigation. Thus, the performance of the method was tested at different pH levels in the slightly acidic medium (2.8-5.0) using an acetate buffer solution. The strongest quenching effect was seen at a pH of 3.5 ± 0.2. Therefore, a pH of 3.5 was used (see Fig. 2) in the recommended procedure.

#### The volume of acetate buffer

**Fig. 3:**
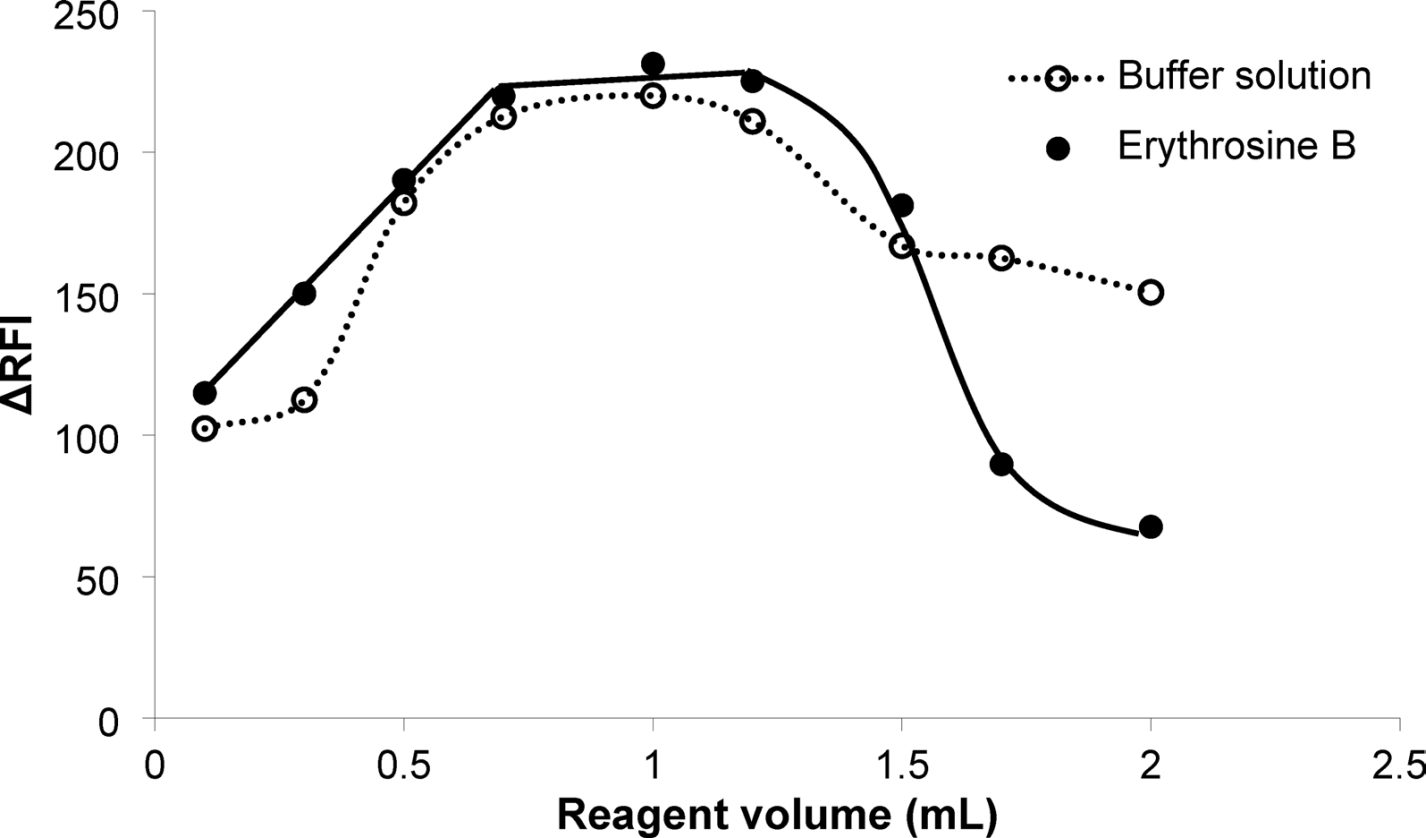
The effect of volume of acetate buffer (··○··) and erythrosine B (0.016% g w/v) (-●-) on the difference in relative fluorescence intensity values of the reaction of product between mirabegron and erythrosine B

The range of volumes of buffer (0.5-3.0 mL) have been tested during the optimization of the proposed method (Fig. 3). It was noticeable that 1.0 mL volume of the acetate buffer was the optimum for getting the maximum quenching value. The use of low volume buffer solution was not suitable owing to its inability to perfectly control the pH of the reaction solution. On the other hand, the use of a higher volume of the buffer solution above the recommended range was also not appropriate. The reason for such low values may be attributed to the high buffer strength leading to effective competition of the acetate anion to bind with the drug cation rather than the dye anion.

#### The volume of erythrosine B

The impact of erythrosine B concentration on the method`s performance was tested using different volumes of the reagent having a fixed concentration (0.016% g w/v). As shown in Fig. 3, the quenching response increased by increasing the reagent volume up to 0.7 mL. The maximum quenching effect was observed when using reagent volumes in the range of 0.7–1.2 mL. Further increases in volume gradually diminish the observed fluorescence quenching value. The low value of the fluorescence quenching apart from the previous range may be attributed to the insufficient amount of the reagent at lower reagent volumes or its self-quenching at higher volumes. Therefore, 1.0 of 0.016% g w/v of erythrosine B was selected as the volume of choice in the present work.

### The stoichiometry and mechanism of the reaction

**Fig. 4:**
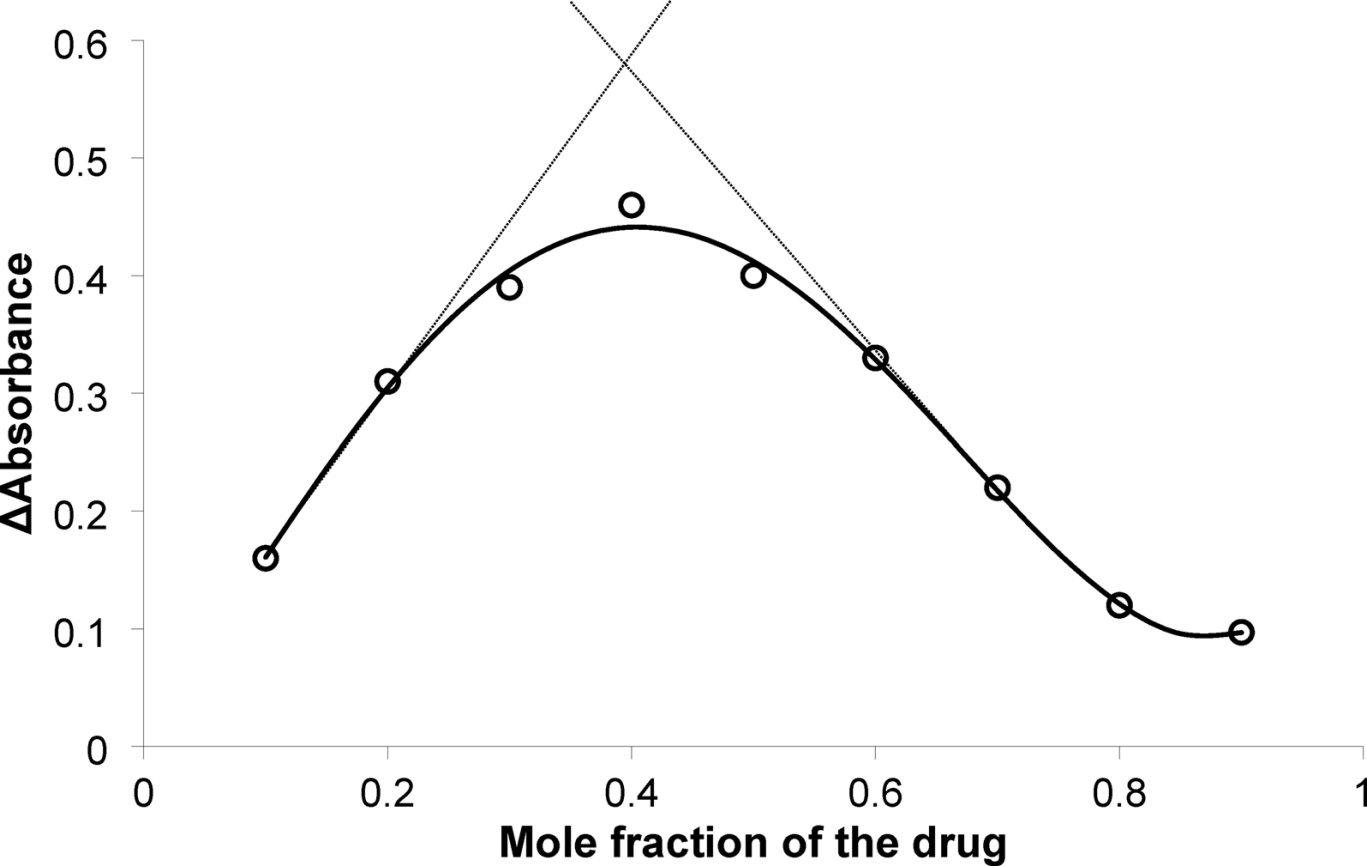
The application of Job’s method for the determination of the stoichiometry of the reaction between mirabegron and erythrosine B using (1.8 × 10 ^*p*−4^ M) solution of both the drug and the dye using the absorbance as a signal

Job’s method of continuous variation was employed spectrophotometrically in purpose to avoid the Inner Filter Effect (IFE) [33] that may happen to determine the ratio between the used dye and the investigated drug. The stoichiometric ratio of the reaction was determined by utilizing solutions of both MBG and erythrosine B having equal molarity (1.8 × 10^− 4^ M). Job's plot was constructed by plotting the corrected absorbance versus the mole fraction of the drug as described in the experimental part. The maximum absorbance has been seen at mole fraction around 0.4, as shown in Fig. 4. This result pointed out that the ratio between MBG and erythrosine B in the reaction was approximately 1:1.

To explain the mechanism of the reaction between the dye and the drug according to the given ratio, it is imperative to first inspect the structure of the dye. The dye has two acidic groups that undergo dissociation in a somewhat acidic environment (pH 3.5 or 4.2), resulting in the formation of a monovalent anion. Even though erythrosine B possesses a carboxylic group, the hydroxyl moiety is accountable for the process of complex formation. Erythrosine B’s molecular structure features two iodine atoms positioned next to the hydroxyl group on the xanthine skeleton. The iodine atoms act as highly electronegative entities that reduce the density of electrons on the oxygen atom that is found on the hydroxyl moiety. This would result in more potent ionization of the hydroxyl moiety which exhibits stronger binding activity than that of the carboxylic moiety located on the phenyl ring [34].

**Fig. 5:**
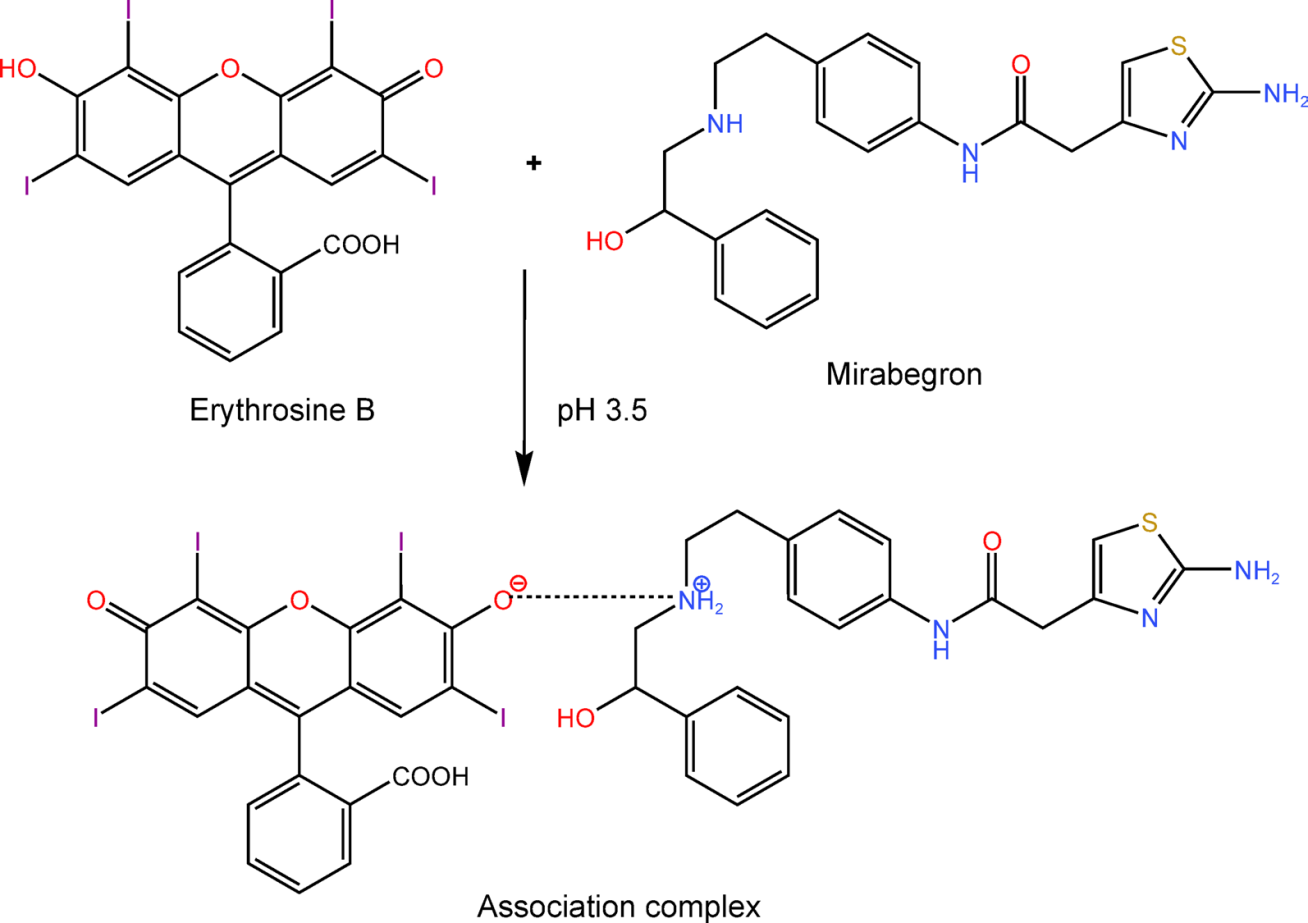
the suggested mechanism of action between mirabegron and erythrosine B

MBG contains two amino groups: a primary amine linked to the thiazole ring and a secondary group in the aliphatic chain bridge. The basicity of the secondary amine is higher than that of the primary amine. The low basicity of the primary amine is due to the electron-withdrawing activity of the thiazole ring and also to its amine-imine tautomerism [35]. The higher basicity of the secondary group enhances the electrostatic interaction between the electron-rich nitrogen atom containing the lone pair and the erythrosine B anion having a negative charge. The previously indicated somewhat acidic environment causes the hydroxyl moiety in erythrosine B to ionize, resulting in the formation of a negatively charged monovalent anion. In this environment, the secondary amine of the drug undergoes protonation, resulting in the formation of the drug cation. Therefore, the negatively charged dye anion and the positively charged drug cation would undergo strong electrostatic attraction in addition to hydrophobic interactions, resulting in the formation of a complex that is 1:1 (Fig. 5).

### Mechanism of fluorescence quenching

There are a couple of pathways that could explain the quenching of erythrosine B`s fluorescence upon its interaction with MBG including; dynamic quenching by collision of molecules, static quenching, transfer of energy, and excited state reactions. To investigate the mechanism of quenching in the present study, Stern–Volmer equation was employed which had been commonly exploited [36–38]. The ratio between the fluorescence of erythrosine B in the presence (F) and absence (F_0_) of MBG was calculated (F and F_0_, respectively) and plotted against the concentration [Q] of the MBG in mole utilizing the next equation [39]:

$${F_o}/F = 1 + {K_{SV}}\left[ Q \right]$$

Where, *K*_SV_ is the Stern–Volmer constant. Stern–Volmer plot show a linear relationship which has an intercept of approximately 1.0. The slope of the calibration line is equal to the Stern Volmer constant (*K*_SV_), which was found to be 2.87 × 10^5^. The quenching rate constant (*k*_q_) can be calculated using the obtained value of *K*_SV_, according to the following equation:

$${K_{{\text{SV}}}}={K_{\text{q}}}{\tau _0}$$

Where; *τ*_0_ is the natural radiation lifetime of erythrosine B which equal 89 ps [40]. By substituting in the previous equation, the quenching rate constant would be 3.22 × 10^12^ L mol^− 1^ s^− 1^. The obtained value is higher than the highest reported quenching value by collision (2 × 10^10^ L mol^− 1^ s^− 1^) [33]. Consequently, it could be obviously confirmed that static quenching is ruling the quenching of fluorescence upon complex formation where the formation occurred between erythrosine B in the ground state and MBG.

### Validation of the studied method

The suggested procedures were validated in accordance with the requirements set by the International Council of Harmonization (ICH). The examined specifications included linearity, accuracy, precision, limits of quantitation and detection, as well as robustness.

#### Linearity as well as limits of detection and quantitation

After setting up the best conditions for the analysis indicated earlier, the calibration curve has been constructed by graphing the ΔRFI versus the of MBG concentrations. It emerged that the values of ΔRFI (Relative Fluorescence Intensity) was proportional to the concentration of MBG within the range of 0.05–1.2 µg/mL. A linear regression analysis using the least-squares approach was performed to get all the validation parameters (Table 1). The coefficient of correlation was found to be 0.9970, demonstrating a high level of linearity of the studied method. The detection and quantitation limits were determined to be 0.01 and 0.04 µg/mL, respectively.

**Table 1:** Statistical parameters for the determination of MBG by the proposed spectrofluorimetric method

Parameters	Value
λ ex (nm)	520
λ em (nm)	550
Linear range (µg\ml)	0.05–1.2
Intercept ± SD	67.02 ± 1.39
Slope ± SD	302.19 ± 2.10
Correlation coefficient (r)	0.999
Determination coefficient (r^2^)	0.999
Limit of detection (LOD, µg\ml)	0.01
Limit of quantitation (LOQ, µg\ml)	0.04

#### Accuracy

The accuracy of the suggested approach has been evaluated using the standard addition method. The Betmiga^®^ tablet solution was analyzed using the proposed technique. Then various quantities of the MBG standard solution (at concentrations of 50%, 100%, and 150% of the tablet concentration) were added to previously analyzed Betmiga^®^ tablet solution and the total content of MBG were determined. The suggested approach demonstrated high accuracy, as evidenced by the good recoveries achieved (96.67 -100.66%) as shown in Table 2.

**Table 2:** Analysis of Betmiga^®^ tablets by the proposed method using standard addition method

Taken concentration (µg\ml)	% Added	Found concentration (µg\ml)	% Recovery* ± SD
0.3	0%	0.295	98.33 ± 1.19
	50%	0.435	96.67 ± 1.98
	100%	0.604	100.66 ± 1.31
	150%	0.741	98.82 ± 1.16

*The value is the average of three determinations (*n* = 3)

#### Precision

To evaluate the intraday and inter-day precision levels, three replicate determinations of three distinct concentrations of MBG standard solutions were utilized through applying the procedure of the proposed approach. The analysis was performed on the same day or in three consecutive days for the two precision levels, respectively. The suggested approach demonstrated great repeatability and reproducibility, as evidenced by the low values of relative standard deviation (RSD) obtained (Table 3).

**Table 3:** The intra- and inter- day precision for determination of MBG by the proposed method

Concentration (µg\ml)	% Recovery * ± % RSD	
	Intra- day precision	Inter-day precision
0.3	102.96 ± 1.29	101.73 ± 0.54
0.5	100.58 ± 1.22	100,36 ± 1.22
0.7	98.65 ± 1.37	99.71 ± 0.36

* The value is the average of three determinations (*n* = 3)

#### Robustness

In order to examine the robustness of the method, it is recommended to examine whether the analytical method is not affected by small changes in its parameters. Thus, minor modifications were implemented to the experimental variables, followed by assessment based on estimating the standard deviation and percentage recovery. Buffer pH, buffer volume, in addition to dye volume, were the parameters that were varied. The results demonstrated the strong resilience of the approach, as it yielded outstanding percentage recoveries with minimal percentage relative standard deviation (RSD) values (Table 4).

**Table 4:** Robustness for determination of MBG by the proposed method

Parameter	Value	% Recovery * ± SD	% RSD
Volume of erythrosine B	0.7 1.0 1.3	103.54 ± 1.56 101.31 ± 0.88 100.73 ± 0.07	1.50 0.87 0.06
pH of buffer	3.2 3.5 3.7	99.76 ± 0.23 100.94 ± 1.01 100.25 ± 0.51	0.23 1.00 0.50
Volume of buffer	0.7 1 1.2	99.83 ± 1.21 99.78 ± 0.24 98.47 ± 1.66	1.21 0.24 1.68

* The value is the average of three determinations (*n* = 3)

### Applications of the proposed method

#### Analysis of mirabegron pharmaceutical tablets

The offered methodology was effectively utilized to analyze MBG in the prepared solution of Betmiga ^®^tablet. Five replicates were conducted using the proposed procedure. After the results were compared to another previously reported method [5] by applying the student’s t-test and variance F test. The calculated values (0.129 and 1.567, respectively) were smaller than the tabulated records (2.306 and 6.338, respectively), indicating no substantial discrepancy between the developed approach and the stated method (Table 5) in respect to accuracy and precision. This proves the ability of the present approach to accurately and precisely quantify MBG in its dosage forms with no notable interfering liability from the tablet`s excipients in these formulations. Thus, the developed method could be an effective alternative for the analysis of the cited drug in quality control units.

**Table 5:** Analysis of tablets containing MBG using the proposed and the reported methods

Parameter	Spectroflourimeteric method	Reported method
% Recovery	99.07	99.73
SD	1.39	1.11
Number of determinations	5	5
t-value^*^	0.129	
F-value	1.567	

* The student t test, the tabulated value is 2.306

#### Application to spiked human plasma

The spectrofluorimetric method’s great sensitivity allows for accurate assessment of MBG in both in vivo and in vitro settings, namely in human plasma, with no interference from plasma components. To confirm that no interference from the components of plasma, a blank experiment was conducted using identical protocols like plasma samples that did not contain MBG. For estimating MBG concentration in vitro study the corresponding regression equation was utilized. The obtained mean recovery value was 97.59 ± 1.82% (Table 6).

**Table 6:** Analysis of MBG in spiked human plasma using the spectrofluorometric method

Concentration (µg\ml)	% Recovery ± SD	% RSD
0.3	95.49 ± 1.88	1.97
0.7	98.68 ± 1.690	1.71
1.2	98.59 ± 0.41	0.41
Mean	97.59 ± 1.82	1.86

* The value is the average of three determinations (*n* = 3)

#### Solution stability

The stability of MBG in biological samples was investigated by spiking plasma samples with two concentrations of MBG (300 and 700 ng/ml), and these samples were stored for 6 h at room temperature, for 48 h at − 20 °C, and exposed to three freeze and thaw cycles. The sample was subjected to freeze at – 20 °C for 24 h then allowed to thaw at room temperature. This cycle was repeated for 3 cycles. After each storage condition, the samples were analyzed using the proposed method in triplicate (the lowest and highest QC concentrations). In addition, the stability samples were analyzed promptly, just prior to preparation. The concentrations of the analytes computed from the stored aliquots were compared with those found in the promptly analyzed samples. Table 7 summarizes the findings from the stability investigation, which indicated good stability of the drug in the solutions under the conditions studied, as seen from the good recovery percentages.

#### Application to real plasma of human

After a fixed daily dose of MBG 100 mg, steady state conditions were reached by seven days with t _max_ of five hours after the last dose administration, and the c_max_ reported was 136 ng/mL [30]. Because of its sensitivity, the suggested fluorescence-based method was utilized to screen the levels of medication in human plasma. Thus, the provided method’s applicability was further assessed by determining the plasma circulation levels of MBG in three healthy human volunteers (25,31, 37 years old). The participants provided written consent and administered two Betmiga ^®^ 50 mg tablets orally for 7 successive days. Blood samples were taken from them after five hours of the last orally taken dose (100 mg MBG). Samples were treated as previously mentioned in spiked samples, and the concentrations of the drug in the real plasma samples were computed using the regression equation constructed in spiked human plasma [ΔRFI = 0.3452 x MBG concentration (ng/mL) ÷ 65.6], *r* = 0.9982. Interestingly, real human plasma samples had MBG content ranges between 125.8 and 134.9 ng/mL. Based on the findings of the present investigation, it was found that the MBG concentration in real human plasma was approximately 130.3 ± 6.1 ng/mL. The C_max_ value found in the present study is in line with the reported value in the published work [30]. According to this information, the spectrofluorimetric method was successfully applied for the determination of MBG in real plasma of human (Table 6). Therefore, the proposed fluorescence-based method may offer a simple substitute for effective MBG quantification in biological samples.

**Table 7:** Stability of MBG at two different concentrations under different storage conditions

Concentration (ng\ml)	% Recovery * ± SD		
	Short-term 6 h at room temp (Bench stability)	Long time freezing for 48 h at – 20 °C	Freeze-thaw (3 cycles at − 20 °C)
300	101.03 ± 1.09	102.27 ± 1.11	101.65 ± 1.15
700	98.97 ± 1.33	98.61 ± 2.79	98.79 ± 1.80

* The value is the average of three determinations (*n* = 3)

### The estimation of the greenness of the proposed experiment

**Fig. 6:**
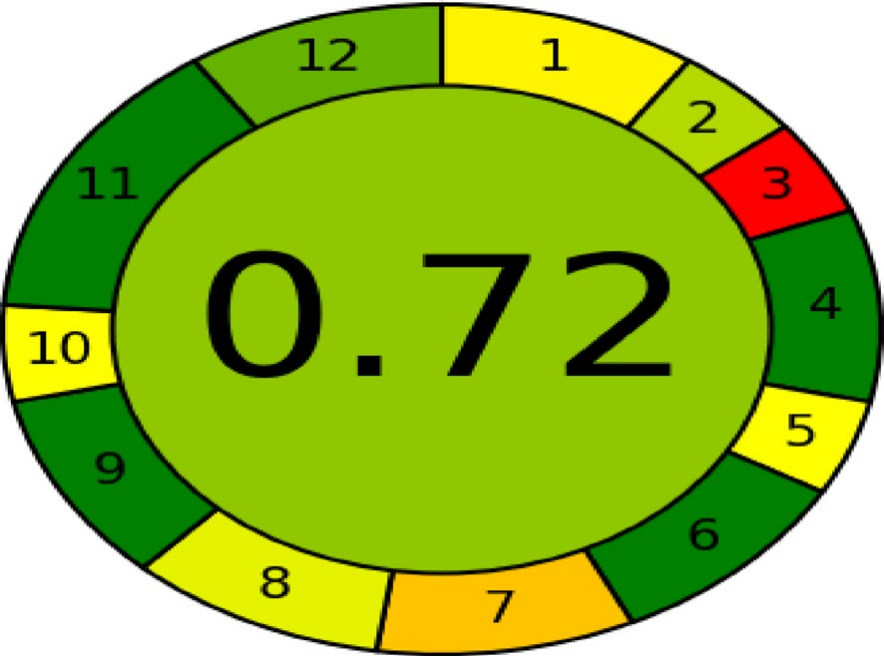
Output of the AGREE method to evaluate the method’s greenness

The environmental friendliness of the proposed analytical methodology was rigorously evaluated using the Analytical Greenness (AGREE) metric [41] which can be considered as a comprehensive tool grounded in the twelve principles of Green Chemistry. Our methodology achieved an AGREE score of (0.72), indicating a strong alignment with green analytical chemistry principles and this is obvious in Fig. 6. Therefore, the AGREE pictogram offers a straightforward visual evaluation of an analytical method’s environmental sustainability, aligning with the 12 principles of Green Analytical Chemistry (GAC).

**Fig. 7:**
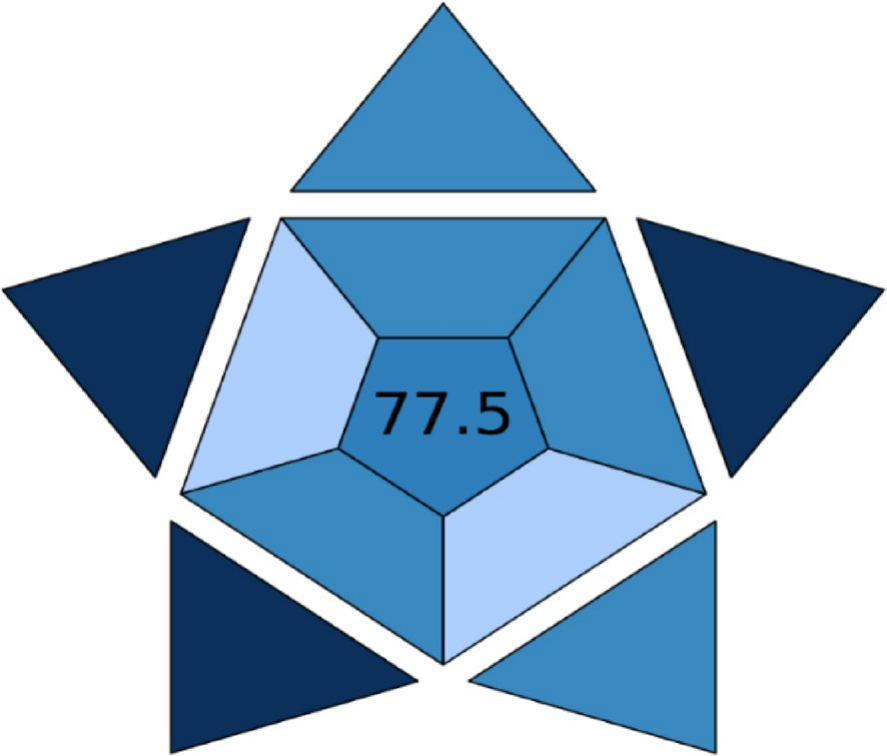
Output of the GABI method to evaluate the method’s blueness

Furthermore, for the purpose of confirmation that this method is experimentally friend the blueness of the proposed experiment has been evaluated by applying BAGI (Blue Applicability Grade Index) method [41] which is BAGI assesses practicality, scoring 0–100 based on cost, time, instrument complexity, and ease of implementation that proves to be a tool for confirming the practicability of the proposed experiment which gives a score of (77.5) and this indicates the very good performance of the proposed spectrofluorimetric method Fig. 7.

#### Comparative study for greenness evaluation

A comparative assessment of greenness evaluation between the proposed approach and published techniques reported just a while ago discloses that the developed spectrofluorimetric method demonstrates superior environmental sustainability compared to existing methodologies. This advantage is confirmed in terms of reduced energy consumption, minimized use of hazardous solvents, lower waste generation, and a higher AGREE score, as delineated in Table 8. The AGREE metric for the spectrophotometric technique [9] was determined to be (0.68), whereas the reported HPLC [21] yielded the lowest score (0.55), attributable to its elevated energy demands. Conversely, the electroanalytical approach [16] method attained a score of (0.62). Notably, the spectrofluorimetric method achieved an AGREE score of (0.72), underscoring its significantly enhanced greenness profile compared to contemporary analytical techniques. This finding substantiates the method’s alignment with the principles of green analytical chemistry over other analytical techniques that have been reported recently.

**Table 8:** The comparison in greenness between the proposed method and other analytical methods

Methodology	Reference	Energy consumption (kWh per sample)	Hazardous solvents	Waste generation (mL/ run)	AGREE score
1—Spectrophotometric technique	[9]	< 0.1	Low (Ethanol)	~ 10 mL	0.68
2—Electroanalytical approach	[16]	4.5 to 5.0	Moderate (orthophosphoric acid, glacial acetic acid, boric acid, and graphite powder)	~ 25	0.62
3—HPLC method	[21]	Between 10 and 20	High (acetonitrile, phosphate)	~ 32	0.55
4—The proposed method		< 0.1	Low (Methanol)	~ 10 mL	0.72

## Conclusion

A novel method for MBG fluorescence detection was developed, which has the advantage of being more appropriate than earlier studies. The method being used is thought to be the first effort to identify MBG in real human plasma using a fluorescent approach. The neoteric technique offered unparalleled advantages in terms of precision, selectivity, eco-friendliness, speed of processing, and convenience. In addition, it does away with the need for sample modification, in contrast to the reported LC methods, which require time-consuming steps and sample pre-treatment. It also does not suffer the drawbacks of published spectrofluorimetic methods, such as the use of several component reagents, the use of very dangerous chemicals, and prolonged heating. The quantification limit was 40.0 ng/mL, and the detection limit was 10.0 ng/mL across a concentration range of 0.05–2.0 µg/mL. The results showed that after 5 h, from the last dose of 100 mg taken orally for 7 days, the concentration of MBG in real human plasma was 130.07 ± 6.1 ng mL. The method suggested here is considered an essential tool for an interference-free bioanalysis of MBG. Therefore, the proposed method provides a useful alternative for studying the pharmacokinetics and bioavailability of MBG as well as medicine quality control without the need for tedious sample extraction or pretreatment steps.

## Data Availability

No datasets were generated or analyzed during the current study.
